# Unveiling the Genetic Diversity and Antimicrobial Resistance Profiles of *Salmonella* Population From 2016 to 2020 in Thai Canal Water

**DOI:** 10.1111/1758-2229.70160

**Published:** 2025-08-01

**Authors:** Jirachaya Toyting‐Hiraishi, Toyotaka Sato, Neunghatai Supha, Yuwanda Thongpanich, Motohiro Horiuchi, Jeewan Thapa, Chie Nakajima, Yasuhiko Suzuki, Fuangfa Utrarachkij

**Affiliations:** ^1^ Division of Bioresources Hokkaido University International Institute for Zoonosis Control Sapporo Japan; ^2^ Laboratory of Veterinary Hygiene, Faculty of Veterinary Medicine Hokkaido University Sapporo Japan; ^3^ Graduate School of Infectious Diseases Hokkaido University Sapporo Japan; ^4^ One Health Research Center Hokkaido University Sapporo Japan; ^5^ Department of Microbiology, Faculty of Public Health Mahidol University Bangkok Thailand; ^6^ International Collaboration Unit Hokkaido University International Institute for Zoonosis Control Sapporo Japan; ^7^ Division of Research Support Hokkaido University Institute for Vaccine Research & Development Sapporo Japan

**Keywords:** antimicrobial resistance, canals, environmental health, *Salmonella*, Thailand, whole genome sequencing

## Abstract

*Salmonella* is one of the important pathogens causing acute gastroenteritis, and antimicrobial‐resistant *Salmonella* raises a critical public health concern. Canals in Bangkok, Thailand, play a vital role as sources of agricultural and daily water usage. By employing whole genome sequencing to analyse 351 *Salmonella* genomes isolated between 2016 and 2020, we expanded the understanding of the characteristics and antimicrobial resistance properties of *Salmonella enterica* found in Bangkok canals, an underrepresented biome in research. *Salmonella* Agona was the dominant serotype, while S. Typhimurium and its monophasic variant were periodically found. Seven new sequence types (STs) were identified, including STs 11,346, 11,347, 11,348, 11,349, 11,350, 11,351, and 11,352. Seven chromosomal‐mediated gene mutations and 50 antimicrobial resistance genes were detected. The three most common resistance genes were *tet*(A), *bla*_TEM‐1B_, and *qnrS1*. The *tet*(X4) was first identified in the *Salmonella* population in Thailand, and *mcr‐3.1* was also detected. In total, 39.0% of the strains were potentially multidrug‐resistant. The strains carried double amino acid substitutions in GyrA and ParC, and a strain with GyrA substitutions and *qnrS1* exhibited the strongest resistance to nalidixic acid and ciprofloxacin. Most of the ceftazidime, ceftriaxone, and cefotaxime‐resistant strains (66.7%) harboured *bla*_CTX‐M‐55_. Col(pHAD28) was the predominant plasmid replicon type. Phylogenetic analysis of *Salmonella* STs 34 and 213 from canal water and the strains from databases showed the possibility of circulation of STs 34 and 213 between canal water and humans in Thailand and worldwide. These findings shed light on the circulation of antimicrobial‐resistant pathogens in the environmental water and advocate for incorporating environmental sampling into comprehensive AMR surveillance programmes within a One Health framework.

## Graphical abstract

By employing whole genome sequencing to analyse 351 *Salmonella* genomes isolated between 2016 and 2020, the characteristics and antimicrobial resistance properties of *Salmonella enterica* found in Bangkok canals were elucidated. Among studied isolates, 39.0% were potentially multidrug‐resistant, and STs 34 and 213 found might have circulated between canal water and humans.

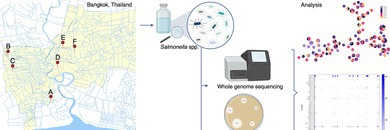

## 1 Introduction

*Salmonella* is accountable for more than 95 million infections, with 50,000 gastroenteritis fatalities globally (James et al. 2018; Roth et al. 2018). In Southeast Asia, nontyphoidal *Salmonella* ranks at the fourth spot of gastroenteritis causes, responsible for over 16 million cases and nearly 16,000 deaths yearly (World Health Organization, Regional Office for South‐East Asia 2016). While traditionally associated with foodborne sources, waterborne *Salmonella* infection is also a considerable risk (Collier et al. 2021; World Health Organization, Regional Office for South‐East Asia 2016). A previous meta‐analysis study examining the publications worldwide reported that the average frequency of *Salmonella* in surface water was 31.97% (Rocha et al. 2022). The frequency and risk might be higher in the warmer climate of a tropical region, where the bacteria can persist and proliferate in the environmental water, as demonstrated in our prior study, where *Salmonella* was present in over 90% of canal water samples from Thailand (Toyting, Supha, et al. 2024).

Antimicrobial resistance (AMR) is an escalating public health threat worldwide driven by the extensive use of antimicrobial agents in humans, livestock, aquaculture, and agriculture. Aquatic environments, including rivers and canals, are likely significant contributors to AMR distribution within society (Amarasiri et al. 2020; Singh et al. 2022; Stanton et al. 2022). Additionally, multidrug‐resistant (MDR) *Salmonella* has been found in diverse environmental water sources globally (Burjaq and Abu‐Romman 2020; Callahan et al. 2019; Kadykalo et al. 2020; Song et al. 2018), implying that antimicrobial‐resistant *Salmonella* could use the aquatic environments as a reservoir. Nevertheless, monitoring AMR in these aquatic environments is frequently neglected compared to human‐focused surveillance, and its dynamics still need to be better understood despite its critical importance (Huijbers et al. 2019; Larsson et al. 2018; Nappier et al. 2020).

Bangkok is the capital and the most populous city of Thailand. As of 2023, Bangkok is home to 16% of the country's population (Central Intelligence Agency 2023). Bangkok has an extensive canal network that functions in various ways, including wastewater and sewage management, drainage, agricultural resources, transportation, tourism, and recreational activities (Jiarakul 2015). Our previous study on genomic characterisation of *Salmonella* from Bangkok canal water revealed that a significant proportion of *Salmonella* strains were potentially MDR, and all strains harboured crucial virulence factors related to salmonellosis. These strains carried diverse plasmid replicon types, suggesting the possible horizontal transfer of AMR genes. Furthermore, the study showed the possible circulation between canal water and food source isolated *S*. Agona in Thailand (Toyting, Nuanmuang, et al. 2024). Despite the initial study being conducted, it was limited by the number of strains analysed. Therefore, more comprehensive research is necessary to fully understand the molecular epidemiology and dynamics of AMR profiles in *Salmonella*, as well as the distribution of AMR through aquatic ecosystems and the role of canals as reservoirs of resistant bacteria in Bangkok, Thailand.

To fill the knowledge gap, this study aimed to provide a comprehensive longitudinal analysis that extends the understanding of the potential threat of AMR, genetic relationship, and antimicrobial susceptibility of *Salmonella* strains from Bangkok canals. By utilising WGS on an extensive collection of *Salmonella* isolates from canal water over 5 years (2016–2020), the present study provides valuable information that will aid in public health risk evaluation and advise response measures in line with the One Health approach to AMR issues in Thailand.

## 2 Materials and Methods

### 2.1 Bacterial Strains

A total of 351 *Salmonella* strains were isolated from canal water samples collected from six distinct canals in Bangkok, Thailand, over 5 years from 2016 to 2020. The sample collection and bacterial isolation protocols were described in the previous study (Toyting, Nuanmuang, et al. 2024). Briefly, the water samples were collected following the World Health Organisations guidelines (World Health Organization 1997). The 50 mL of water samples were mixed with an equal volume of buffer peptone water in the pre‐enrichment step and incubated overnight at 37°C. The enriched samples underwent selective enrichment by being dropped onto Modified Semi‐Solid Rappaport‐Vassiliadis (MSRV) agar (Becton, Dickinson and Company, Franklin Lakes, NJ) and incubated at 42°C for 24 h. Then, the swarming edge on the MSRV agar was picked and streaked onto Xylose Lysine Deoxycholate (XLD) agar (Becton, Dickinson and Company) and incubated overnight at 37°C. Subsequently, three *Salmonella* colonies were randomly picked from the XLD agar plates for biochemical confirmation using Triple Sugar Iron Agar and Motility Indole Lysine Medium. After the *Salmonella* strains were biochemically identified, they were preserved in Luria‐Bertani (LB) broth (Becton, Dickinson and Company) supplemented with 30% glycerol and stored at −80°C for further analysis. Bacterial species identification was performed using Speciator on the Pathogenwatch platform, explained in the *In silico species confirmation* section.

### 2.2 DNA Extraction and Whole Genome Sequencing

*Salmonella* genomic DNA was extracted using the phenol‐chloroform method. First, crude DNA was mixed with 2% SDS and proteinase K, then incubated at 56°C for 1 h. After incubation, an equal volume of phenol/chloroform/isoamyl alcohol (25:24:1) was added, then vortexed to form an emulsion, and centrifuged at 15,000 rpm for 5 min. The upper aqueous phase was transferred to a new tube, mixed with an equal volume of chloroform, vortexed, and centrifuged again. The upper phase was then transferred, and DNA was precipitated with 3 M sodium acetate and 99.5% ethanol, incubated at −80°C for 30 min, and centrifuged at 15,000 rpm for 20 min. The DNA pellet was washed with 80% ethanol, centrifuged, air‐dried, and resuspended in TE buffer. The DNA quality and concentration were assessed using a NanoDrop spectrophotometer and a Qubit fluorometer, respectively. For each isolate, 1 ng of genomic DNA was used to prepare sequencing libraries with the Nextera XT DNA Library Preparation Kit (Illumina Inc., San Diego, CA), following the manufacturer's guidelines. Library fragment sizes were evaluated using a Bioanalyzer (Agilent Technologies). *Salmonella* strains were sequenced as multiplexed libraries on the Illumina MiSeq platform, using the manufacturer's protocol for 600 cycles to produce 300 bp paired‐end reads.

### 2.3 Genome Assembly and Quality Checking

The whole genome sequences of *Salmonella* strains from canal water were uploaded to the Bacterial and Viral Bioinformatics Resource Center (https://www.bv‐brc.org/) for quality checking and genome assembly. First, the quality of raw reads was checked by FastQC version 0.12.1 (Babraham Bioinformatics 2016), and the sequencing adapters were trimmed away by Trim Galore version 0.6.5 (https://www.bioinformatics.babraham.ac.uk/projects/trim_galore/). The genome assembly was performed using SPAdes version 3.13.0 (Bankevich et al. 2012), and the quality of the assemblies was assessed using QUAST version 5.2.0 (Gurevich et al. 2013).

### 2.4 In Silico Species Confirmation, Serotyping, Sequence Typing, Plasmid Replicon Typing, and Antimicrobial Resistance Gene Identification

The assembled genomes were uploaded to Pathogenwatch (https://pathogen.watch/) for species confirmation using Speciator (Argimón et al. 2021; Ondov et al. 2016). *Salmonella In Silico* Typing Resource (SISTR) was employed to predict serotypes (Yoshida et al. 2016). The sequence type (ST) of each strain was determined using multilocus sequence typing (MLST) with a *Salmonella*‐specific scheme (Jolley et al. 2018). The strains with novel allelic profiles were submitted to EnteroBase (https://enterobase.warwick.ac.uk/) for the new ST assignment on May 27, 2024 (Achtman et al. 2020; Alikhan et al. 2018). The minimum spanning tree based on MLST was visualised by PHYLOViZ 2.0 (Nascimento et al. 2017). ResFinder (https://cge.food.dtu.dk/services/ResFinder/) (Bortolaia et al. 2020; Camacho et al. 2009; Zankari et al. 2017) was used to identify both types of AMR determinants, namely acquired resistance genes and chromosomal‐mediated gene mutations. Plasmid replicon type was categorised by Inctyper, an in‐house tool of Pathogenwatch that uses BLAST and PlasmidFinder database to identify the contigs containing an Inc. reference gene (Argimón et al. 2021; Camacho et al. 2009; Carattoli et al. 2014).

### 2.5 Phylogenetic Analysis

All S. Typhimurium (Strains MU45, MU61, MU173, and MU354) and its monophasic variant (*Salmonella* I 1,4,[5],12:i:‐; Strains MU70, MU171, MU179, MU252, MU282, MU306, MU339, MU371, and MU375) isolated from canal water were uploaded to EnteroBase to construct a maximum‐likelihood tree. This tree was based on non‐repetitive core SNPs (minimum presence 95%) and was generated by RAxML (Stamatakis 2014) through the EnteroBase SNP Project Dendrogram Module (Zhou et al. 2020). A total of 113 isolates from Thailand, identified as S. Typhimurium and *Salmonella* I 1,4,[5],12:i:‐, were searched in EnteroBase and included in the analysis (Table S2). Isolates lacking information on the isolation source and year were excluded from the analysis. *Salmonella* I 1,4,[5],12:i:‐ Strain MU282 was used as a reference for this analysis. A global phylogenetic analysis of the predominant ST (ST34) among S. Typhimurium and its monophasic variant from canal water was conducted, incorporating 190 *Salmonella* ST34 strains from EnteroBase (Table S3), with *Salmonella* I 1,4,[5],12:i:‐ Strain MU282 serving as the reference. Additionally, a global phylogenetic analysis of S. Typhimurium ST213 was performed, including 199 *Salmonella* strains with ST213 from EnteroBase (Table S4). *Salmonella* Strain MU354 was used as the reference for the ST213 analysis.

### 2.6 Antimicrobial Susceptibility Testing

Antimicrobial susceptibility testing by disk diffusion method (Clinical and Laboratory Standards Institute 2020) for nalidixic acid (NAL) and ciprofloxacin (CIP) was performed in a prior study (Toyting, Supha, et al. 2024). Additionally, antimicrobial susceptibility testing using marketed antimicrobial disks (Oxoid, England) against ceftazidime (CAZ) (30 μg), ceftriaxone (CRO) (30 μg), and cefotaxime (CTX) (30 μg) was conducted in this study using the same method described in the previous study (Toyting, Supha, et al. 2024). The antimicrobial susceptibility of the *Salmonella* strains was assessed by measuring the inhibition zone diameters according to *Enterobacteriaceae* breakpoints and classified as susceptible, intermediate, or resistant. The inhibition zone diameter breakpoints for CAZ were sorted as ≥ 21 mm, 18–20 mm, and ≤ 17 mm for susceptible, intermediate, and resistant, respectively. For CRO, the breakpoints were determined as ≥ 23 mm, 20–22 mm, and ≤ 19 mm for susceptible, intermediate, and resistant, respectively. The breakpoints for CTX were labelled as ≥ 26 mm, 23–25 mm, and ≤ 22 mm for susceptible, intermediate, and resistant, respectively (Clinical and Laboratory Standards Institute 2020).

### 2.7 Data Visualisation

The graphical representations of the AMR determinant and plasmid replicon type results were generated by GraphPad Prism version 10.2.3 for macOS (GraphPad Software, Boston, MA), and the detailed results are shown in Table S1. The resulting phylogenetic trees were visualised using iTOL (Letunic and Bork 2019). The graphical abstract was created by Biorender (https://www.biorender.com).

## 3 Results

### 3.1 Species Confirmation and Serotype Prediction of *Salmonella* From Bangkok Canal Water

All the strains were confirmed as *Salmonella enterica* . In total, 60 serotypes were identified (Table 1), with serotype Agona (17.7%, *n* = 62/351) being the most common serotype, followed by serotype Rissen (8.5%, *n* = 30/351) and serotype Stanley (6.8%, *n* = 24/351). Additionally, serotype Typhimurium, serotype I 1,4,[5],12:i:‐ (a monophasic variant of serotype Typhimurium), and serotype Paratyphi B biovar Java were occasionally found. Serotypes Agona, Rissen, Stanley, and Tennessee were discovered in all six canals.

**TABLE 1:** Distribution of 351 *Salmonella* isolates among different serotypes, STs, and sampling sites.

Serotype	ST	No. of isolates						
		Sampling sites						Total
		Canal A	Canal B	Canal C	Canal D	Canal E	Canal F	
Aberdeen	426	1	1	1	0	1	1	5
Agona	13	5	9	14	9	12	13	62
Altona	1549	0	0	1	0	0	0	1
Amsterdam	590	0	0	2	0	1	2	5
Anatum	64	1	0	1	4	2	1	9
Apeyeme	1546	0	1	0	1	1	2	5
	10,000	0	1	0	0	0	0	1
Bareilly	203	0	0	0	0	1	0	1
Bovismorbificans	1499	0	0	1	0	1	1	3
Braenderup	22	0	0	0	2	1	0	3
Brandenburg	65	0	0	0	0	1	0	1
Chester	343	1	0	0	0	0	0	3
	2063	2	0	1	0	0	0	1
Corvallis	1541	1	0	2	5	2	5	15
Derby	40	3	0	0	0	1	1	5
Dublin	74	0	0	0	0	1	0	1
Emek	76	0	1	2	1	2	1	7
Farmsen	2812	1	0	0	0	0	0	1
	11,352	1	0	0	0	0	0	1
Gaminara	2152	1	0	0	0	0	0	1
Give	516	0	0	1	1	3	0	5
Haifa	11,347	0	0	0	0	1	0	1
Hvittingfoss	446	5	2	1	1	0	1	10
I 1,4,[5],12:b:—	423	0	0	1	1	3	0	5
I 1,4,[5],12:i:—	34	0	2	2	3	2	0	9
I B:f,g,s:e,n,z15	13	0	0	0	0	0	1	1
I G:‐:l,w	592	0	0	0	0	1	0	1
Indiana	2040	1	0	0	0	0	0	1
Isangi	1994	0	1	0	0	0	0	1
Javiana	1547	0	0	1	0	1	0	2
Kedougou	1543	0	0	0	0	1	0	1
Kentucky	198	0	1	0	2	0	0	3
	696	0	1	0	0	0	0	1
Kiambu	309	0	0	0	0	1	0	1
Langensalza	1799	0	0	0	0	1	0	1
	3157	0	1	0	0	1	0	2
Lexington	1542	0	0	0	2	1	0	3
Livingstone	543	0	0	1	1	0	0	2
	11,346	0	0	0	0	2	1	3
Matopeni	2456	1	0	1	0	0	2	4
Mbandaka	413	3	0	1	2	4	5	15
Mgulani	2033	0	1	0	0	0	0	1
Molade	544	0	1	0	0	0	0	1
	2657	0	0	1	0	0	1	2
Montevideo	1531	0	0	0	0	1	0	1
Mountpleasant	287	6	0	0	0	0	0	6
Muenchen	82	0	0	0	1	0	1	2
Muenster	321	0	1	1	0	3	2	7
Newport	31	1	1	0	0	0	0	2
	46	1	0	3	0	0	0	4
Panama	48	0	1	0	1	0	1	3
Paratyphi B var. Java	43	2	0	0	0	0	0	2
Poona	1069	1	0	0	0	0	1	2
	3236	2	0	0	0	1	2	5
Ramatgan	11,349	1	0	0	0	0	0	1
Rissen	469	2	6	6	6	6	4	30
Schwarzengrund	96	0	0	0	0	1	0	1
	11,350	0	0	1	0	0	0	1
Senftenberg	14	0	0	1	0	1	0	2
	210	0	0	0	0	1	1	2
Singapore	462	0	1	1	1	0	0	3
	501	0	1	0	1	0	0	2
Stanley	29	0	4	3	5	5	4	21
	2299	1	0	0	1	0	0	2
	11,351	0	0	0	0	0	1	1
Tennessee	319	1	1	3	2	1	4	12
Thompson	2417	0	0	1	0	0	0	1
Typhimurium	34	0	0	1	0	0	0	1
	36	0	0	1	0	0	1	2
	213	0	1	0	0	0	0	1
Uganda	684	0	2	0	0	3	1	6
Urbana|Neudorf	512	1	0	0	0	0	1	2
Virchow	16	0	0	0	1	0	0	1
	359	0	0	2	1	1	4	8
Wandsworth	1498	0	0	0	0	1	0	1
Waycross	1419	0	1	0	0	0	0	1
Weltevreden	365	1	0	2	0	0	4	7
	11,348	0	0	0	0	1	0	1
Total		47	43	61	55	75	70	351

### 3.2 Sequence Types of *Salmonella* From Bangkok Canal Water

**FIGURE 1:**
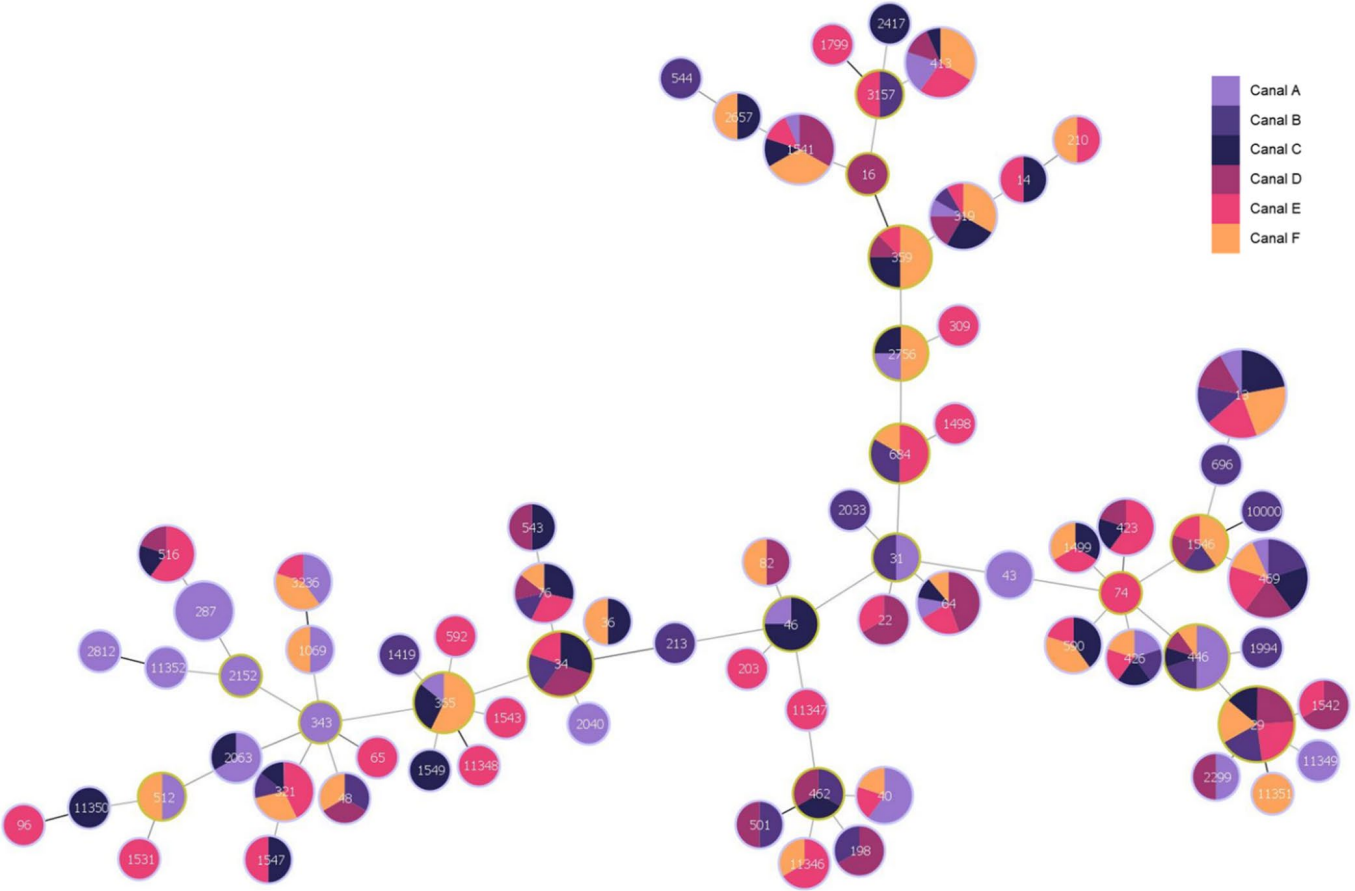
Minimum spanning tree based on MLST data for the 351 *Salmonella* isolates analysed in this study. Each circle on the tree represents a single sequence type (ST), with the ST number displayed in small print at the centre of the circle. The size of each circle corresponds to the number of strains within that ST. The colours within the circles represent the specific canal from which the isolates were obtained.

In total, 76 STs were identified (Table 1). The most common ST was ST13 (17.9, *n* = 63/351), followed by ST469 (8.5%, *n* = 30/351) and ST29 (6.0%, *n* = 21/351). Eleven *Salmonella* strains from canal water were assigned new STs, including STs 11346, 11,347, 11,348, 11,349, 11,350, 11,351, and 11,352. All seven new STs with their allelic profiles are depicted in Table 2. Figure 1 shows STs 13, 319, and 469 dispersed in all six canals. ST11346 showed a relation to ST462 that also linked to ST11347 and ST46. ST11348 was related to ST355, while ST11349 and ST11351 were associated with ST29. ST11350 showed a relation with ST96 and ST512. ST11352 was related to ST2812 and ST2152. S. Typhimurium strains from canal water belonged to 3 STs, including ST34, ST36, and ST213, while its monophasic variant strains only belonged to ST34.

**TABLE 2:** *Salmonella* strains with new STs identified in this study.

Strain	Year	Canal	Serotype	ST	*aroC*	*dnaN*	*hemD*	*hisD*	*purE*	*sucA*	*thrA*
MU309	2020	E	Livingstone	11,346	137	2	36	2012	2	7	6
MU310	2020	F	Livingstone	11,346	137	2	36	2012	2	7	6
MU358	2020	E	Livingstone	11,346	137	2	36	2012	2	7	6
MU203	2018	E	Haifa	11,347	5	14	21	2013	6	12	17
MU319	2020	E	Weltevreden	11,348	130	97	25	125	84	1474	101
MU69	2016	A	Ramatgan	11,349	14	1415	108	33	5	12	8
MU41	2016	C	Schwarzengrund	11,350	43	47	49	2014	41	15	3
MU147	2017	F	Stanley	11,351	1661	16	20	18	8	12	18
MU149	2017	A	Farmsen	11,352	514	384	38	16	1642	35	410

### 3.3 Antimicrobial Resistance Determinants and Phenotypes of *Salmonella* From Bangkok Canal Water

**FIGURE 2:**
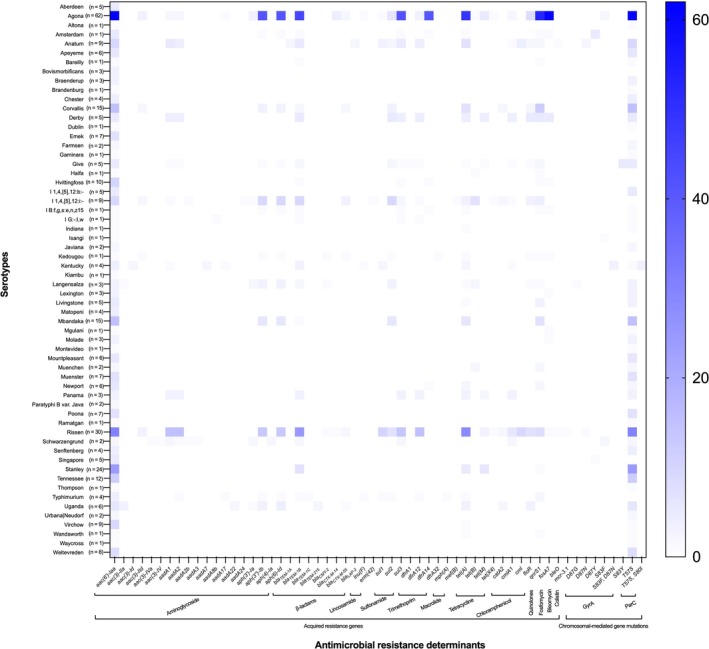
Number of the isolates harbouring antimicrobial resistance determinants. The *Y*‐axis represents serotypes, and the *X*‐axis represents the antimicrobial resistance determinants. The colour gradient represents the number of strains.

As illustrated in Figure 2, a total of 50 acquired resistance genes were identified by ResFinder. These genes confer resistance to 12 distinct classes of antimicrobials comprising aminoglycoside [*aac(6′)‐Iaa*, *aac(3)‐Id*, *aac(3)‐IId*, *aac(3)‐Iva*, *aac(3)‐IV*, *aadA1*, *aadA2*, *aadA2b*, *aadA3*, *aadA7*, *aadA8b*, *aadA17*, *aadA22*, *aadA24*, *aph(3′)‐Ia*, *aph(3″)‐Ib*, *aph(4)‐Ia*, and *aph(6)‐Id*], β‐lactams (*bla*_TEM‐1A_, *bla*_TEM‐1B_, *bla*_TEM‐1C_, *bla*_TEM‐215_, *bla*_CMY‐2_, *bla*_CTX‐M‐14_, *bla*_CTX‐M‐55_, *and bla*_LAP‐2_), lincosamide [*Inu*(F) and *erm*(42)], sulfonamide (*sul1*, *sul2*, and *sul3*), trimethoprim (*dfrA1, dfrA12, dfrA14*, and *dfrA32*), macrolide [*mph*(A) and *mef*(B)], tetracycline [*tet*(A), *tet*(B), *tet*(M), and *tet*(X4)], chloramphenicol (*catA2*, *cmlA1*, *cml*, and *floR*), quinolones (*qnrS1*), fosfomycin (*fosA7*), bleomycin (*bleO*), and colistin (*mcr‐3.1*). All the strains possessed *aac(6′)‐Iaa*, a cryptic gene in *Salmonella* strains. When excluding *aac(6′)‐Iaa*, at least one acquired resistance gene was detected in 54.1% (*n* = 190/351) of the study strains. The three most prevalent resistance genes were *tet*(A), *bla*_TEM‐1B_, and *qnrS1*, found in 36.8% (*n* = 129/351), 33.3% (*n* = 117/351), and 31.6% (*n* = 111/351) of the strains, respectively. Co‐harbouring of *bla* and *qnr* genes was identified in 19.9% (*n* = 70/351) of the studied strains, in which the co‐carriage of *bla*_TEM‐1B_ and *qnrS1* was the majority (82.9%, *n* = 58/70), followed by the combination of *bla*_TEM‐1B_, *bla*_CTX‐M‐55_, and *qnrS1* (11.4%, *n* = 8/70). *tet*(X4) was detected in three strains, including *S*. Agona MU243, *S*. Rissen MU317, and *Salmonella* I 1,4,[5],12:i:‐ MU371. In addition, *mcr‐3.1* was detected in one *S*. Rissen strain (Strain MU36) along with the resistance genes against aminoglycoside [*aac(3)‐Id*, *aph(3″)‐Ib*, and *aph(6)‐Id*], β‐lactams (*bla*_TEM‐1B_ and *bla*_CTX‐M‐55_), sulfonamide (*sul2*), tetracycline [*tet*(A)], chloramphenicol (*catA2, floR*), and quinolones (*qnrS1*).

Seven chromosomal‐mediated gene mutations related to fluoroquinolone resistance were identified in *gyrA* and *parC* via *in silico* analysis by ResFinder, as depicted in Figure 2. The amino acid substitutions in GyrA were found at positions 83 [from serine to phenylalanine or tyrosine: S83F (2.3%, *n* = 8/351) or S83Y (1.4%, *n* = 5/351)] and 87 [from aspartic acid to glycine or asparagine or tyrosine: D87G (0.3%, *n* = 1/351) or D87N (1.7%, *n* = 6/351) or D87Y (1.7%, *n* = 6/351)]. The amino acid substitution in ParC at position 57 from threonine to serine (T57S) was the most prevalent mutation detected in 79.5% (*n* = 279/351) of the strains. The amino acid substitution in ParC at position 80 from serine to isoleucine (S80I) was also identified (0.9%, *n* = 3/351). Notably, double amino acid substitutions in both positions of GyrA (S83F, D87N) and ParC (T57S, S80I) were observed in only *S*. Kentucky ST198 strains (Strains MU152, MU153 and MU235).

Twenty‐three *Salmonella* strains from canal water harboured chromosomal‐mediated gene mutations in GyrA, and all were resistant to NAL. Among these 23 strains, seven were resistant to NAL and CIP. Three strains carried double amino acid substitutions in both positions of GyrA and ParC, and another strain carried an amino acid substitution in GyrA, and *qnrS1* had the lowest inhibition zone diameter (Table 3). In part of β‐lactams resistance, the strains exhibited resistance to CTX, CRO, and CAZ which were 6.0% (*n* = 21/351), 5.7% (*n* = 20/351), and 4.3% (*n* = 15/351), respectively. Twelve strains were resistant to all three drugs, with 66.7% (*n* = 8/12) carrying *bla*_CTX‐M‐55_ (Table 4).

**TABLE 3:** NAL and CIP‐resistant *Salmonella* strains with resistance determinants.

Strain	Serotype	Canal	Year	Diameter of antibiotic disc (mm)		Chromosomal‐mediated gene mutations		PMQR genes
				NAL^a^	CIP^b^	GyrA	ParC	*qnrS*
MU50	Langensalza	E	2016	≤ 6	20	D87G	T57S	+
MU152	Kentucky	D	2017	≤ 6	12	S83F, D87N	T57S, S80I	−
MU153	Kentucky	D	2017	≤ 6	12	S83F, D87N	T57S, S80I	−
MU178	Give	E	2018	≤ 6	20	S83Y	T57S	−
MU235	Kentucky	B	2018	≤ 6	12	S83F, D87N	T57S, S80I	−
MU275	Agona	E	2019	≤ 6	12	S83F	T57S	+
MU281	Give	E	2019	≤ 6	19	S83Y	T57S	+

^a^ Breakpoints for NAL; *S* ≥ 19, *I* = 14–18, *R* ≤ 13.

^b^ Breakpoints for CIP; *S* ≥ 31, *I* = 21–30, *R* ≤ 20.

**TABLE 4:** CAZ, CRO, and CTX‐resistant *Salmonella* strains with resistance determinants.

Strain	Serotype	Canal	Year	Diameter of antibiotic disc (mm)			β‐lactamase genes		
				CAZ^a^	CRO^b^	CTX^c^	*bla*_TEM‐1B_	*bla*_CMY‐2_	*bla*_CTX‐M‐55_
MU36	Rissen	B	2016	8	≤ 6	≤ 6	+	−	+
MU50	Langensalza	E	2016	15	17	≤ 6	−	+	−
MU77	Kedougou	E	2016	11	≤ 6	≤ 6	−	+	+
MU121	Corvallis	D	2017	17	≤ 6	≤ 6	−	−	−
MU171	I 1,4,[5],12:i:—	D	2018	16	≤ 6	≤ 6	+	−	+
MU178	Give	E	2018	11	≤ 6	≤ 6	−	−	−
MU193	Corvallis	C	2018	16	≤ 6	≤ 6	+	−	+
MU197	Corvallis	E	2018	16	≤ 6	≤ 6	+	−	+
MU208	Rissen	B	2018	≤ 6	≤ 6	≤ 6	−	−	+
MU264	Agona	D	2019	16	≤ 6	≤ 6	+	−	+
MU282	I 1,4,[5],12:i:—	E	2019	16	≤ 6	≤ 6	+	−	+
MU317	Rissen	B	2019	15	17	21	+	+	−

^a^ Breakponits for CAZ; *S* ≥ 21, *I* = 18–20, *R* ≤ 17.

^b^ Breakpoints for CRO; *S* ≥ 23, *I* = 20–22, *R* ≤ 19.

^c^ Breakpoints for CTX; *S* ≥ 26, *I* = 23–25, *R* ≤ 22.

**FIGURE 3:**
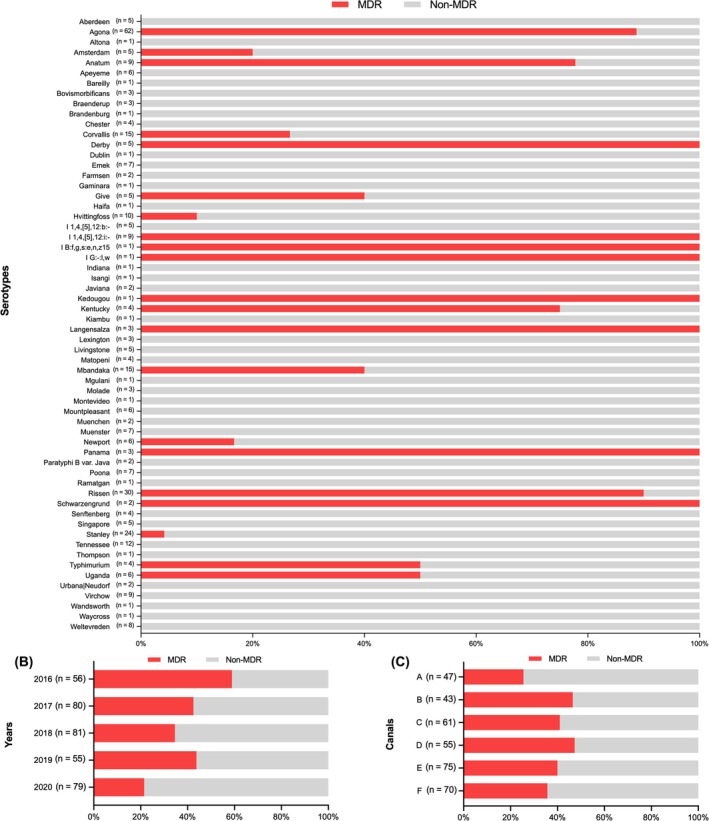
Percentage of MDR strains in (A) each serotype, (B) each year, and (C) each canal categorised by possession of acquired resistance genes and chromosomal‐mediated gene mutations, incorporated with antimicrobial susceptibility results. Red columns represent MDR strains, and grey columns represent non‐MDR strains.

MDR *Salmonella* in this study was defined as a *Salmonella* strain carrying the resistance mechanisms in ≥ 3 classes of antibiotics. Since *aac(6′)‐Iaa* is the cryptic gene in *Salmonella*, possessing this gene was not considered. The strains harbouring ParC T57S but did not exhibit resistance to NAL and CIP from antimicrobial susceptibility testing were determined as non‐resistant to quinolones. When considering the combination of acquired resistance genes, chromosomal‐mediated gene mutations, and phenotypic results, 39.0% (*n* = 137/351) of the strains were potentially MDR. Among the 60 identified serotypes, 21 exhibited MDR characteristics. Notably, there were several serotypes where 100% of the strains were MDR, including Derby, I 1,4,[5],12:i:‐, I B:f,g,s:e,n,z15, I G:‐,w, Kedougou, Langensalza, Panama, and Schwarzengrund (Figure 3A). As illustrated in Figure 3B, the proportion of MDR *Salmonella* strains from Bangkok canal water decreased from 58.9% in 2016 to 42.5% in 2017, then increased to 45.5% in 2019 and decreased again to 21.5% in 2020. Regarding the canals, as shown in Figure 3C, Canal D and Canal B had the highest proportions of MDR *Salmonella* strains, with 47.3% (*n* = 26/55) and 46.5% (*n* = 20/43), respectively. In contrast, Canal A had the lowest proportion at 25.5% (*n* = 12/47).

### 3.4 Plasmid Replicon Types of *Salmonella* From Bangkok Canal Water

**FIGURE 4:**
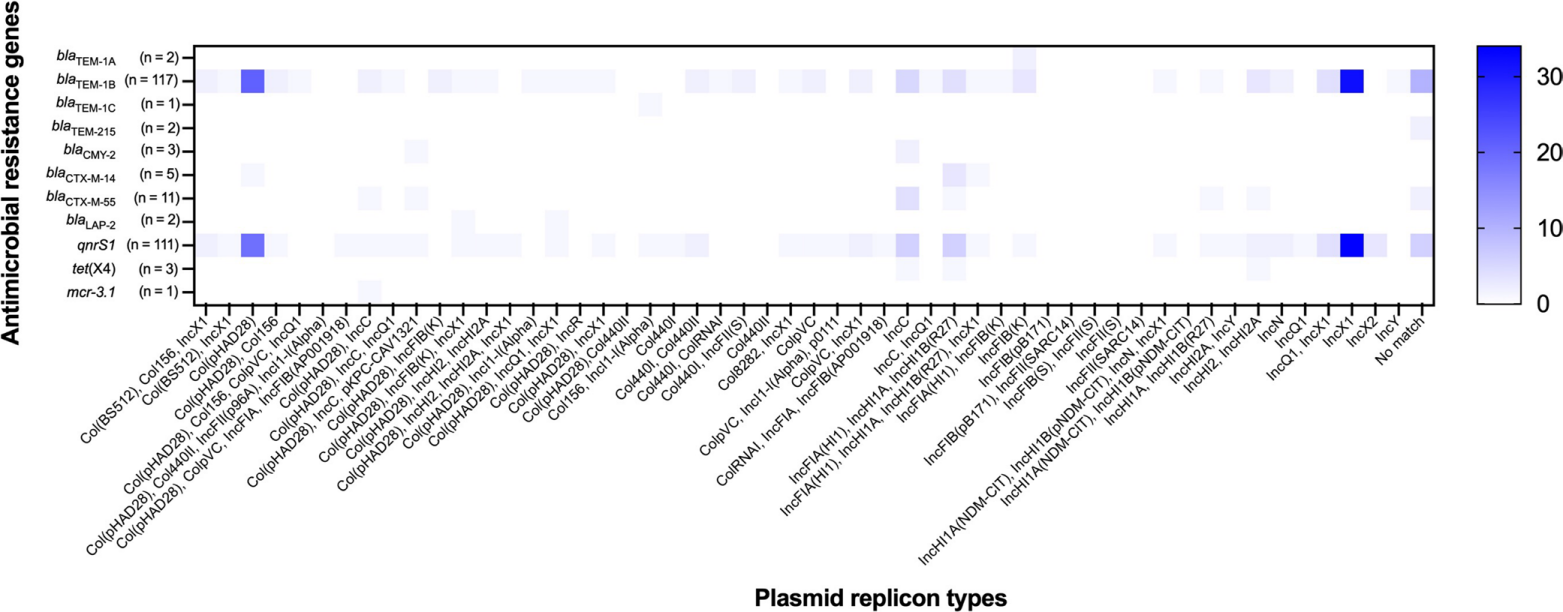
Number of the strains harbouring acquired resistance genes and plasmid replicon types. The *Y*‐axis represents the acquired resistance genes against β‐lactams, fluoroquinolones, tetracycline, and colistin. The *X*‐axis represents the plasmid replicon types. The colour gradient represents the number of strains.

Over half (58.4%, *n* = 205/351) of *Salmonella* strains isolated from Bangkok canal water were positive for the sequence of plasmid replicons (Table S1). In total, 52 plasmid replicon harbouring patterns were identified, in which the strain processing only Col(pHAD28) was predominant, followed by the strains harbouring IncX1 alone. A small number of the study strains harboured four plasmid replicon types. Focusing on extended‐spectrum β‐lactamase (ESBL), plasmid‐mediated quinolone resistance (PMQR), and mobile colistin resistance genes, the most common resistance genes, *bla*_TEM‐1B_ and *qnrS1*, were found along with various plasmid replicon types. Noteworthy, two strains were positive for *bla*_TEM‐1A_, and both carried IncFIB(K). All three strains that possessed *bla*_CMY‐2_ and *qnrS1* also had IncC. Two strains harboured *bla*_LAP‐2_ and *qnrS1*, which also carried Col(pHAD28) and IncX1. Nearly all strains with *bla*_CTX‐M‐55_ and *qnrS1* were positive for either IncC or IncHI1/2. Three strains harbouring *tet*(X4) possessed different plasmid replicon types with the following patterns: [1] IncHI2 and IncHI2A, [2] IncC, and [3] IncFIA(HI1), IncHI1A, and IncHI1B(R27). A strain carrying *mcr*‐3.1, *bla*_TEM‐1B,_ *bla*_CTX‐M‐55_, and *qnrS1* harboured Col(pHAD28) and IncC (Figure 4).

### 3.5 Phylogenetic Analysis of S. Typhimurium and *Salmonella* I 1,4,[5],12:I:‐ From Bangkok Canal Water

**FIGURE 5:**
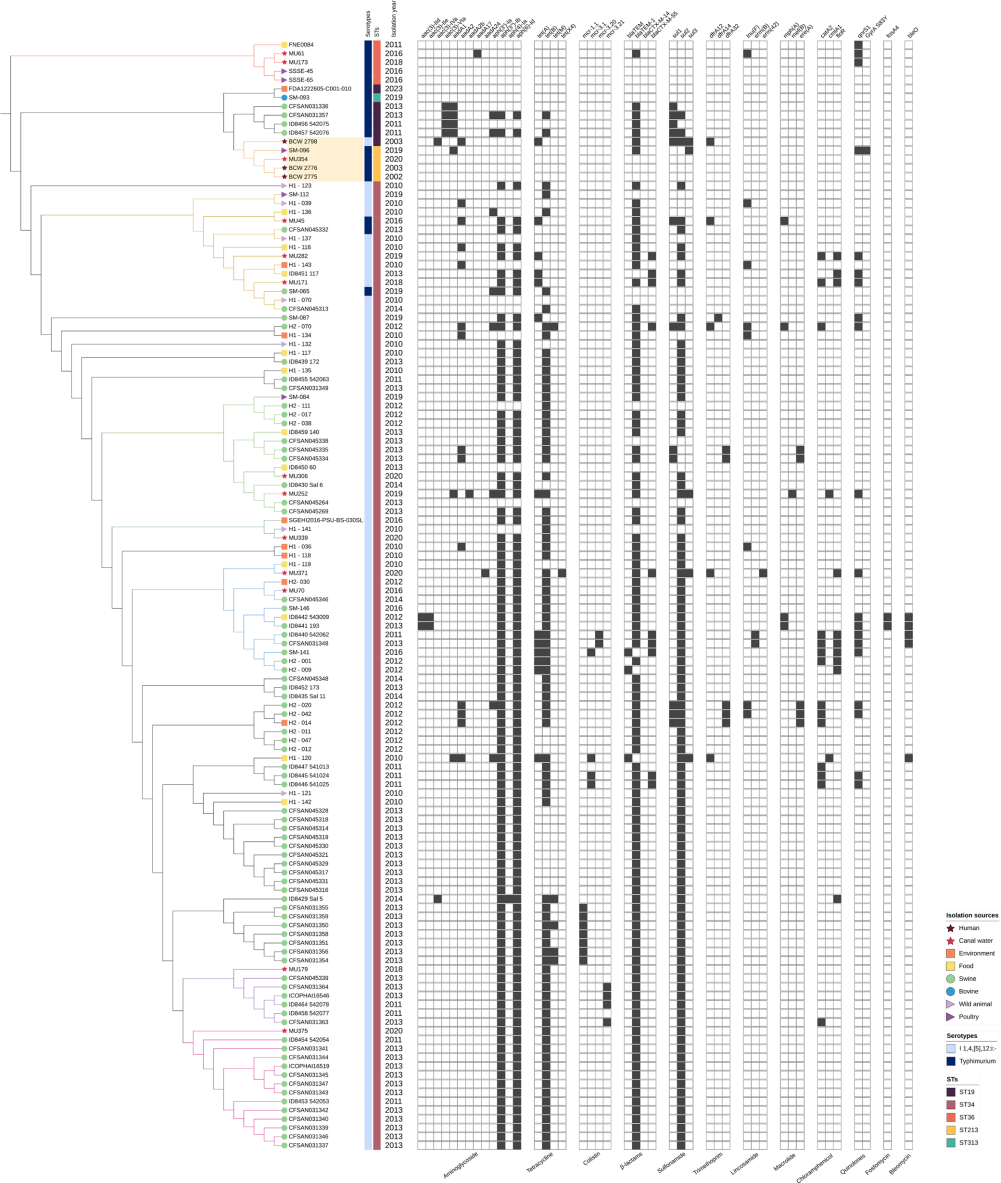
SNP‐based phylogenetic tree of S. Typhimurium and *Salmonella* I 1,4,[5],12:I:‐ from Bangkok canal water and 113 strains from Thailand. The sources of the isolates are denoted by different branch symbols. The serotypes and STs are indicated by distinct colours according to the figure's scheme, as shown in the first and second colour strips. The MDR patterns are displayed in a heatmap, with black indicating gene presence and white indicating gene absence. The clades containing the study strains are marked with coloured branches, with the clade that includes study strains alongside human strains highlighted in yellow.

In the phylogenetic analysis of S. Typhimurium and its monophasic variant isolated from canal water, the strains were compared with those from Thailand and clustered by serotype and ST (Figure 5). The strains within the same cluster also tended to share similar patterns of antimicrobial resistance genes. *Salmonella* I 1,4,[5],12:i:‐ strains from canal water primarily clustered with swine, environmental, and food strains. S. Typhimurium strains MU61 and MU173, which belong to ST36, formed a distinct cluster with environmental and poultry strains. S. Typhimurium strain MU45, part of ST34, was grouped with *Salmonella* I 1,4,[5],12:i:‐ strains MU171 and MU282, as well as isolates from poultry, swine, wild animals, food, and the environment. Notably, S. Typhimurium strain MU354, belonging to ST213, was clustered with strains from poultry and humans.

**FIGURE 6:**
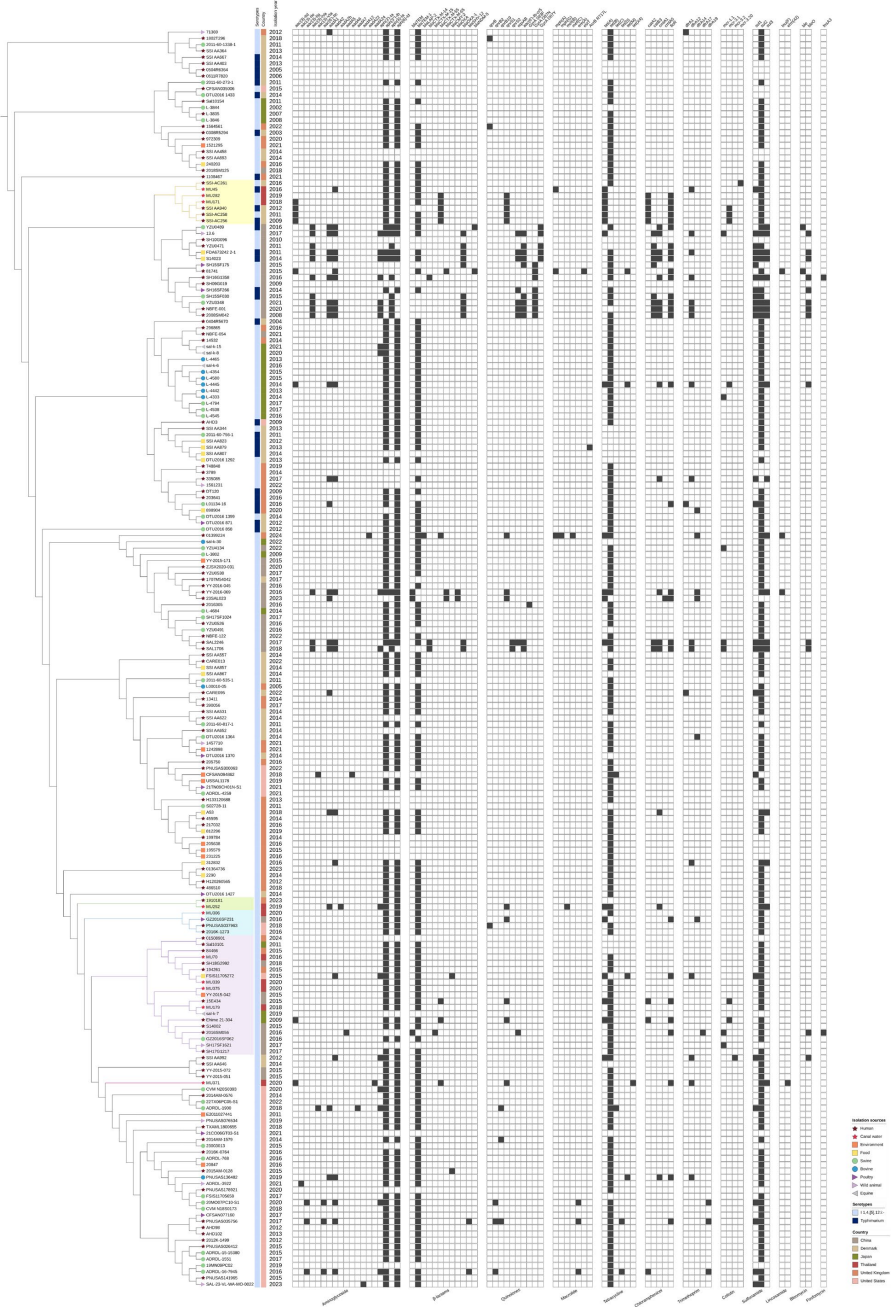
SNP‐based phylogenetic tree of 10 *Salmonella* ST34 from Bangkok canal water and 190 strains from the global collection. The sources of the isolates are represented by different branch symbols. The serotypes and countries of origin are indicated by different colours according to the figure's scheme, shown in the first and second colour strips. The MDR patterns are displayed in a heatmap, with black indicating the presence and white indicating the absence of genes. The clades containing the study strains are highlighted in yellow, green, blue, and purple.

**FIGURE 7:**
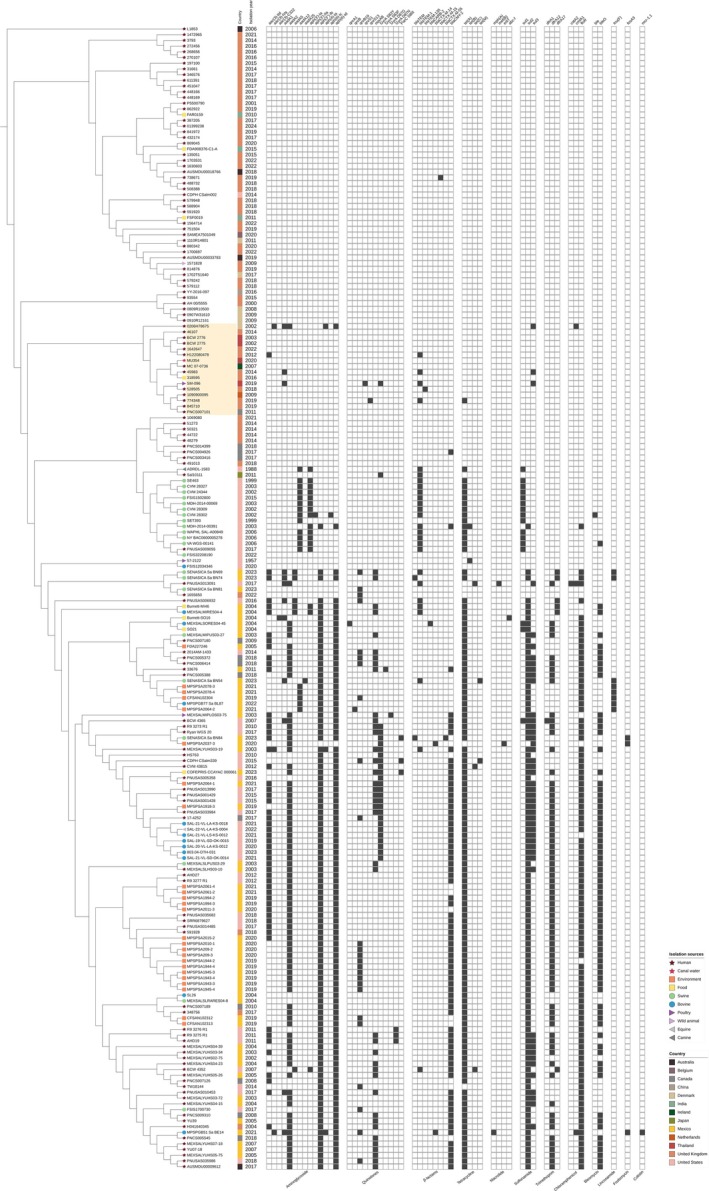
SNP‐based phylogenetic tree of the *Salmonella* ST213 from Bangkok canal water and 199 strains from the global collection. The sources of the isolates are denoted by various branch symbols. The countries of origin are represented by different colours in the figure's colour strip. MDR patterns are illustrated in a heatmap, with black indicating gene presence and white indicating gene absence. The clade that includes the study strains is highlighted in yellow.

The analysis of ST34 strains from canal water alongside a global collection revealed several notable clusters (Figure 6). *Salmonella* strains MU45, MU171, and MU282 were clustered with human strains from Denmark. *Salmonella* strain MU252 was grouped with a human strain from the United Kingdom. *Salmonella* strain MU306 was clustered with a poultry strain from Denmark and human strains from the United States. Additionally, *Salmonella* strains MU70, MU179, MU339, and MU375 were grouped together, forming a cluster with human strains from China, Japan, and the United Kingdom, as well as a food strain from the United States, an equine strain from Japan, and environmental, swine, and poultry strains from China. Further analysis of the ST213 strain from canal water, compared with a global collection, revealed that *Salmonella* strain MU354 clustered with human strains not only from Thailand but also China, Denmark, Ireland, and the United Kingdom (Figure 7).

## 4 Discussion

This study found that *S*. Agona was the dominant serotype in Bangkok canals, which aligned with the previous report (Toyting, Nuanmuang, et al. 2024). S. Typhimurium and its monophasic variant (*Salmonella* I 1,4,[5],12:i:‐) were also detected in canal water. S. Typhimurium is reported as one of the serotypes frequently isolated from human salmonellosis (Hendriksen et al. 2011). Nevertheless, *Salmonella* I 1,4,[5],12:i:‐ has been observed as a new *Salmonella* serotype with rapid emergence worldwide. In the past two decades, it has become one of the most common serotypes causing infections in humans and animals (Sun, Wan, et al. 2019). Furthermore, *Salmonella* I 1,4,[5],12:i:‐ has been reported as a cause of waterborne outbreaks in the United States and Central Italy related to a community's private water system and irrigation water channels, respectively (Cito et al. 2016; Kozlica et al. 2010). The detection of these *Salmonella* serotypes in canal water in the current study underlines the potential risk of *Salmonella* infections associated with Bangkok's waterways.

Seven new STs were identified in the present study, including STs 11346, 11,347, 11,348, 11,349, 11,350, 11,351, and 11,352. This indicates the genetic variation of the *Salmonella* population in Bangkok canals. ST34 was related to several *Salmonella* serotypes, including one S. Typhimurium strain and nine *Salmonella* I 1,4,[5],12:i:‐ strains. This pattern of association has been reported previously in China for *Salmonella* strains isolated from retail meat and meat products (Yang et al. 2019), indicating a broader trend in the genetic variation of *Salmonella* ST34 across different environments and regions.

Mechanisms of fluoroquinolone resistance in *Enterobacteriaceae* include [1] chromosomal mutations in DNA topoisomerase‐encoding genes (*gyrA*, *gyrB*, *parC*, and *parE*) at quinolone‐binding sites known as quinolone‐resistance determining regions (QRDRs), and [2] plasmid‐mediated quinolone resistance (PMQR) genes (*qnrA*, *qnrB*, *qnrC*, *qnrD*, *qnrS*, *qepA*, *aac(6′)‐Ib‐cr*, and *oqxAB*). In the present study, ParC T57S was the most frequently detected amino acid substitution, similar to those reports in China (Chen et al. 2024; Ma et al. 2020) and Korea (Lee et al. 2021). This mutation has been hypothesised as a naturally occurring compensatory mutation (Eaves et al. 2004) and seems to contribute to enhanced bacterial fitness rather than protection against fluoroquinolones (Chang et al. 2021). The double amino acid substitutions in both positions of GyrA (S83F, D87N) and ParC (T57S, S80I) were observed in only three *S*. Kentucky that belong to ST198, all of which were resistant to CIP. These three *S*. Kentucky also exhibited the lowest inhibition zone diameters against CIP, suggesting an elevated resistance level to fluoroquinolones. The genetic background of these CIP‐resistant *S*. Kentucky strains has been confirmed as a piecemeal phenomenon involving successive mutations in *gyrA* and *parC* that are crucial to achieving a high level of CIP resistance (Le Hello et al. 2011). The characteristic of CIP‐resistant *S*. Kentucky is a single amino acid substitution in GyrA, classically S83F, or double amino acid substitutions, S83F, and D87G/N/Y, combined with single or double amino acid substitutions in ParC (Shaheen et al. 2021). The presence of double amino acid substitutions in GyrA and ParC in CIP‐resistant *S*. Kentucky from this study has been reported exclusively in the epidemic clone of *S*. Kentucky ST198 (Wasyl et al. 2015). More worrisome, one *S*. Agona strain carrying amino acid substitutions in GyrA and *qnrS1* also exhibited the lowest inhibition zone diameter against CIP, suggesting co‐carriage of mutations in QRDRs and PMQR gene confers a high level of fluoroquinolone resistance. As isolates from canal water present increased levels of fluoroquinolone resistance, it may complicate bacterial infection treatment and is of great concern.

Regarding the acquired resistance gene to quinolones, *qnr*S1 was detected in a significant proportion of the study strains (31.6%, *n* = 111/351). The *qnrS* gene has become the predominant PMQR gene in *Salmonella* isolated from various sources in Thailand, including humans, pig farms, slaughterhouses, and canals (Eiamsam‐Ang et al. 2022; Sriyapai et al. 2021; Toyting, Nuanmuang, et al. 2024; Toyting, Supha, et al. 2024; Utrarachkij et al. 2017). One possibility is that the plasmids harbouring *qnrS* have low fitness costs relative to other genes, which may confer a competitive advantage under environmental selective pressure (Kaplan et al. 2015; Machuca et al. 2014). Significant concentrations of fluoroquinolones were previously detected in the Thai city canals (Suzuki et al. 2021), which may be considered a selective pressure accelerating the wide distribution of *qnrS* in the *Salmonella* population in the canals. Nevertheless, the actual mechanism of *qnrS* during the transmission needs to be further investigated. Two strains exhibited resistance to CAZ, CRO and CTX without detectable resistance genes. These discrepancies between phenotypic resistance and genetic determinants require further analysis to elucidate the underlying mechanisms.

Colistin is a final therapeutic option for bacterial infections when other antibiotics fail. Nevertheless, it is ranked among the top 10 antimicrobials used in food‐producing animals and medicated feed for food‐producing animals in Thailand, where the consumption rate has continuously increased annually (Health Policy and Systems Research on Antimicrobial Resistance (HPSR‐AMR) Network 2024). In this study, one *S*. Rissen strain isolated in 2016 (Strain MU36) tested positive for *mcr‐3.1*, a gene linked to colistin resistance. In addition, this strain also carried resistance genes against β‐lactams (*bla*_TEM‐1B_ and *bla*_CTX‐M‐55_) and fluoroquinolones (*qnrS1*). The *mcr‐3* gene was initially discovered in China back in 2015 from swine *Escherichia coli* (Yin et al. 2017). However, it was detected in clinical *Salmonella* strains in 2007 from the retrospective study investigating the clinical *Salmonella* strains isolated during 2005–2007 in Thailand (Luk‐in et al. 2021). Hence, *mcr‐3* may have already spread in Thailand since 2007. The co‐occurrence of *mcr‐3.1*, *bla*_CTX‐M‐55_, and *qnrS1* was identified in human *Salmonella* isolates in Thailand (Luk‐in et al. 2021) and Denmark, where the patients had a travel history to Thailand and Vietnam (Litrup et al. 2017). In addition, an earlier report discovered that the Thai canal *S*. Schwarzengrund strain also co‐carried these resistance genes (Toyting, Nuanmuang, et al. 2024).

Tigecycline is considered a last‐resort antibiotic for treating severe infections caused by highly resistant bacteria, including those resistant to carbapenems and colistin (Fang et al. 2020). However, the plasmid‐mediated tigecycline resistance gene *tet*(X4) encodes tetracycline‐inactivating flavoenzymes, which degrade all tetracyclines, including tigecycline (Sun, Chen, et al. 2019). In Thailand, *tet*(X4) was previously reported only in *E. coli* (Li et al. 2023; Yue et al. 2023). Hence, this study marks the first *tet*(X4) report on the *Salmonella* population in the Thai aquatic environment. Taken together with our previous study, the presence of *Salmonella* strains harbouring a wide array of resistance genes in environmental water is concerning, signifying that canal water could potentially be a reservoir for disseminating the AMR genes.

*Salmonella* has been widely distributed in Thai canals, as previously reported (Toyting, Supha, et al. 2024). Several factors may contribute to this, including (1) the stagnant nature of the canals combined with the high population density in Bangkok's urban areas; (2) poor sanitation in some households, the presence of livestock farming, and insufficient wastewater treatment; and (3) Thailand's high levels of precipitation and warm water temperatures. Overall, the physical characteristics of the canals, surrounding land utilisation, human activity, and climatic conditions may create favourable conditions for *Salmonella* proliferation, promote bacterial contamination and distribution, and ultimately contribute to its high prevalence in Thai canals.

A significant proportion of the studied strains were potentially MDR, in which all the strains in several serotypes were MDR. As mentioned previously, *Salmonella* I 1,4,[5],12:i:‐ is one of the most commonly isolated serotypes from humans with rapid spread globally. All *Salmonella* I 1,4,[5],12:i:‐ found in this study were MDR. The antimicrobial resistance patterns in this serotype signify an adaptive advantage, contributing to the evolutionary success of this serotype and its clones (Sun, Wan, et al. 2019). Phenotypically, *S*. Schwarzengrund strains in this study exhibited resistance to NAL and reduced susceptibility to CIP. NAL resistance and reduced susceptibility to CIP were commonly observed in *S*. Schwarzengrund isolated from humans and chicken meat in Thailand (Aarestrup et al. 2007). Genotypically, studied *S*. Schwarzengrund strains were MDR. MDR *S*. Schwarzengrund has been isolated from various sources in Thailand (Niyomdecha et al. 2016; Sirichote et al. 2010). Collectively, MDR *Salmonella* strains have been widely distributed in Thailand, including in environmental water. The percentage of MDR *Salmonella* strains from Bangkok canals increased in 2019 and decreased in 2020. The reduction could be linked to behavioural changes brought about by the COVID‐19 pandemic, including fewer hospital appointments, compliance with social distancing guidelines, a rise in telemedicine, and travel restrictions (Centers for Disease Control and Prevention 2022).

The prevalence of MDR *Salmonella* might be related to the land utilisation plan of the surrounding areas of the canals. In this study, the highest prevalence of MDR *Salmonella* strains was in Canal D and Canal B, while the lowest one was in Canal A. Canal D is in a densely populated residential area, while Canal A is in a sparsely populated residential zone. The contributions of population density and human activities to the diverse antimicrobial resistance gene distribution levels have been previously described (Davis et al. 2020; Jantharadej et al. 2022; Toyting, Nuanmuang, et al. 2024). Canal B is in an agricultural area where most residents are engaged in farming, aquaculture, and livestock (Thawi Watthana Subdistrict Administrative Organization 2018). Aminoglycosides, tetracyclines and quinolones are regularly used in plant production (Miller et al. 2022), while macrolides, tetracyclines and sulfonamides are commonly used in food‐producing animals in Thailand (Lekagul et al. 2023). This frequent antibiotic usage may apply selective pressure on bacterial populations, promoting the survival and multiplication of resistant strains. Furthermore, agricultural practices often involve using animal manure as fertiliser, which can introduce antimicrobial‐resistant bacteria to water systems.

In this study, *bla*_CTX‐M‐55_ was found in the strains carrying either IncC or IncHI1/2 plasmids. A similar finding was reported in a previous study, where *bla*_CTX‐M‐55_ was associated with IncA/C2 or IncHI2 plasmids in *Salmonella* strains from fish and pork (Nadimpalli et al. 2019). IncA/C plasmid is a self‐transferable, broad host range plasmid, which played a vital role in *bla*_CTX‐M‐55_ distribution among blood‐isolated S. Choleraesuis, causing a high prevalence of extended‐spectrum cephalosporin resistance in Thailand. These *bla*_CTX‐M‐55_‐positive strains from canal water harbouring IncC or IncHI1/2 were also positive for *qnrS1*. The co‐existence of ESBL and PMQR genes in *Salmonella* strains suggests a co‐selection mechanism, facilitating the spread of resistance to both cephalosporins and fluoroquinolones (Zhang et al. 2019) in the aquatic environment. IncA/C and IncFIA‐HI1A‐HI1B were previously reported to be involved in *tet*(X4) dissemination in *Enterobacterales* of swine origin (Wang et al. 2022), which is in accordance with the current study. Additionally, a studied strain harbouring *mcr‐3.1*, *bla*_CTX‐M‐55_ and *qnrS1* was detected with IncC. According to a previous study, IncA/C plasmid could possibly be a significant channel of *mcr‐3* distribution in Thailand since 2007 (Luk‐in et al. 2021). The co‐harbouring of PMQR, ESBL, mobile colistin resistance genes, and tigecycline resistance genes, along with the presence of plasmids in *Salmonella* strains from canal water, indicates a risk of horizontal transfer of AMR genes, posing a significant public health threat.

*Salmonella* ST34, initially linked to gastrointestinal infections in Europe, has now emerged as a globally distributed pandemic clone (Cadel‐Six et al. 2021; Petrovska et al. 2016). The analysis of *Salmonella* ST34 strains from Bangkok canal water revealed clustering with human strains from various foreign countries, all of which were MDR. Similarly, *Salmonella* ST213, associated with both gastrointestinal and invasive infections (Serrano‐Fujarte et al. 2024), was previously reported in surface waters utilised in agriculture in Mexico (Ballesteros‐Nova et al. 2022). *S. typhimurium* Strain MU354, which belongs to ST213, was clustered with human strains from Thailand and foreign countries. This genetic relationship between *Salmonella* ST213 from canal water and human strains suggests that S. Typhimurium ST213 might circulate between the aquatic environment and humans in Thailand. Furthermore, the clustering of *Salmonella* ST34 and ST213 strains from canal water with strains from other countries indicates that antimicrobial‐resistant bacteria could be introduced to or spread from Thailand via international trading, travelling, and transportation. These findings also highlighted the potential of *Salmonella* strains for transmission through multiple routes. The observed genetic relatedness between MDR *Salmonella* strains from canal water in Thailand and those from foreign nations accentuates that AMR is not merely a national but an international issue. This eventually emphasises the need for sustainable countermeasures for AMR mitigation and global health protection.

## 5 Conclusion

The capital city of Thailand, Bangkok, features an extensive canal network that serves various functions for the city. Antimicrobial‐resistant *Salmonella* in these canals is concerning and raises a substantial public health threat. This study represents the comprehensive genomic surveillance of *Salmonella* strains from Bangkok canals over 5 years. Out of 351 strains, 137 (39.0%) were found to be MDR. Seven new STs were identified, indicating a wide genetic variation of the *Salmonella* population in Bangkok canal water. Various plasmid types were identified, which could facilitate the horizontal transfer of AMR genes. Further analysis suggested a possible circulation of *Salmonella* ST34 and ST213 between canal water and humans. These findings highlight the role of the aquatic environment in urban areas as a reservoir of MDR *Salmonella*. The information gained from this study helps understand the emergence and dissemination of AMR, facilitating sustainable intervention strategy development.

## Author Contributions

**Jirachaya Toyting‐Hiraishi:** methodology, data curation, investigation, formal analysis, visualization, resources, writing – original draft, software. **Toyotaka Sato:** validation, supervision, writing – review and editing. **Neunghatai Supha:** resources, methodology, validation, writing – review and editing. **Yuwanda Thongpanich:** methodology, validation, resources, writing – review and editing. **Motohiro Horiuchi:** supervision, validation, writing – review and editing. **Jeewan Thapa:** validation, writing – review and editing, supervision, methodology. **Chie Nakajima:** conceptualization, writing – review and editing, funding acquisition, supervision. **Yasuhiko Suzuki:** conceptualization, data curation, investigation, validation, supervision, funding acquisition, visualization, project administration, writing – review and editing, resources. **Fuangfa Utrarachkij:** conceptualization, investigation, validation, supervision, data curation, visualization, resources, project administration, writing – review and editing.

## Conflicts of Interest

The authors declare no conflicts of interest.

## Supporting information


**Table S1:** Three hundred fifty‐one *Salmonella* strains isolated from canal water in Bangkok, Thailand, between 2016 and 2020.

**Table S2:** S. Typhimurium and *Salmonella* I 1,4,[5],12:i:‐ strains from Thailand obtained from the Enterobase database that were included in the phylogenetic analysis.

**Table S3:** *Salmonella* ST34 strains obtained from the Enterobase database that were included in the phylogenetic analysis.

**Table S4:** *Salmonella* ST213 strains obtained from the Enterobase database that were included in the phylogenetic analysis.

## Data Availability

The raw sequences of 351 *Salmonella* strains from Bangkok canal water samples were deposited in the NCBI Sequence Read Archive under the Bioproject number PRJNA1123420.
